# Investigation of the Lived Experience of Family Members of a Person With Intellectual Disabilities Who Presents With Pica

**DOI:** 10.1111/jar.70268

**Published:** 2026-06-17

**Authors:** Natasha Pearson, Peter Baker, Jill Bradshaw

**Affiliations:** ^1^ Kent County Council Folkestone England; ^2^ Tizard Centre University of Kent Canterbury Kent England; ^3^ Health and Social Care (Intellectual Disabilities Research Institute (IDRIS) University of Birmingham Birmingham England

## Abstract

**Background:**

Pica, the persistent ingestion of non‐nutritive substances, poses significant health risks for individuals with intellectual disabilities yet remains under‐recognised, particularly in home settings.

**Method:**

This qualitative study explored the lived experiences of family members supporting a person with intellectual disabilities who engages in pica. Using Interpretative Phenomenological Analysis, four in‐depth, semi‐structured interviews were conducted with primary caregivers.

**Results:**

Five superordinate themes were identified: *Living on High Alert*, *Systemic Failures in Care*, *Emotional Erosion*, *Resourceful Parenting*, and *The Silence Around Pica*. Caregivers described constant vigilance, emotional exhaustion, and the strain of managing life‐threatening behaviours with little professional input. They also demonstrated creativity and resilience while fulfilling multiple roles as carers, advocates, and problem‐solvers.

**Conclusions:**

Findings highlight a profound gap between the severity of pica and the adequacy of professional responses, underscoring the need for increased awareness, training, and specialist support.

## Lay Summary

- Pica is a behaviour where people eat non‐food items such as stones, fabric, or paint. It can be dangerous and sometimes life‐threatening. Pica is more common in people with intellectual disabilities, but it remains poorly understood, and families often manage it with little support.
- This study explored the experiences of family members caring for someone with an intellectual disability who engages in pica. Four parents took part in‐depth interviews.
- Families described living in constant vigilance to keep their loved one safe. Daily life was dominated by fear of choking or poisoning, exhaustion, and the loss of ordinary routines. Parents felt professionals often lacked knowledge about pica, dismissed their concerns, or gave unrealistic advice. This left families feeling isolated and forced to manage complex needs without adequate support.
- Despite these challenges, families showed creativity and resilience, developing practical ways to reduce risks and sharing ideas with others. However, this often came at an emotional and financial cost. Many described the mental strain of never being able to relax.
- Pica was described as a ‘silent’ issue—rarely discussed, even among professionals. Families wanted greater understanding, specialist training, and clearer support pathways.
- This research highlights a major gap between the risks families face and the help they receive. Recognising families' expertise and listening to their experiences could improve safety, reduce stress, and lead to better care for people who engage in pica.

## 1 Introduction

Pica, defined as the persistent consumption of non‐nutritive, non‐food substances for at least one month at a developmentally inappropriate age, is commonly observed in individuals with intellectual disabilities (American Psychiatric Association 2013). Prevalence estimates of pica in people with intellectual disabilities range between 4% and 26% depending on context, severity, and environmental factors (American Psychiatric Association 2013). Pica can lead to serious medical complications including gastrointestinal obstructions, infections, poisoning, and fatal outcomes (Doumbia et al. 2020; Hagopian et al. 2011).

### 1.1 Clinical Interventions and Limitations

Function‐based behavioural interventions from Applied Behaviour Analysis are consistently identified as the most effective treatment for pica (Matson et al. 2013; McAdam et al. 2004). Reviews show multicomponent approaches significantly reduce pica across populations and settings (Hagopian et al. 2011; Moline et al. 2021). However, the evidence base relies heavily on single‐case designs, limiting generalisability and highlighting a lack of large‐scale controlled trials. Pharmacological and nutritional interventions show limited, inconsistent effects and are typically adjunctive rather than standalone (Matson et al. 2013). Practice has shifted from aversive methods to reinforcement‐based, least‐restrictive approaches aligned with positive behaviour support.

Despite progress, more rigorous and scalable research is needed. These interventions require specialised expertise, on‐going monitoring, and intensive resources, which are difficult to replicate outside clinical settings (McKendry and Baker 2018). While effective in institutional contexts, families often lack equivalent support, training, and infrastructure, leaving them to manage high‐risk behaviour in less controlled environments with limited assistance.

### 1.2 Impact on Caregivers

Within the broader literature on caregiving in intellectual and developmental disabilities (IDD), elevated levels of psychological distress, including stress, anxiety, and depression, are well established (Alay and Kaçan 2024; Hayes and Watson 2013). However, caregiving in the context of pica may represent a qualitatively distinct form of burden, characterised not only by intensity but by chronic exposure to risk and uncertainty. Existing models of caregiver burden, which often emphasise cumulative stress and adaptive coping, may not fully capture the sustained hypervigilance required when behaviours present immediate and potentially life‐threatening consequences (Call et al. 2015; Hagopian et al. 2011; McConnell and Savage 2015). In this sense, pica may be more appropriately conceptualised within frameworks of chronic crisis caregiving, where the distinction between acute and on‐going stress becomes blurred.

The requirement for near‐constant supervision disrupts not only sleep and daily functioning but also caregivers' capacity for psychological recovery, contributing to chronic fatigue and sustained anxiety (Hodapp 2007; Schulz and Sherwood 2008). Over time, this persistent state of alertness may align more closely with trauma‐related processes than with traditional stress models. Emerging evidence suggests that caregivers of individuals with complex needs may experience symptoms consistent with complex post‐traumatic stress disorder, driven by repeated exposure to perceived or actual threats (Baker et al. 2021; McConnell and Savage 2015). From a trauma‐informed perspective, this raises important questions regarding how services conceptualise and respond to caregiver need, particularly given that distress may be rooted not only in caregiving demands but in cumulative experiences of crisis and perceived lack of safety.

Financial and occupational impacts further compound this burden, yet these are often under‐theorised within the literature. Rapanaro et al. (2008), described participants' reductions in employment and economic instability; however, these impacts may also be understood as structurally mediated outcomes rather than individual consequences. That is, caregiving‐related financial strain is not solely a function of care demands but reflects broader systemic limitations in the availability of flexible employment, adequate respite provision, and accessible specialist services. Notably, reluctance to utilise respite care due to concerns regarding staff competence (Bigby and Beadle‐Brown 2018) illustrates a critical tension between service availability and perceived safety, further reinforcing caregiver isolation.

Although social support is widely recognised as a protective factor in caregiver resilience (Aldersey 2012), caregivers managing pica may experience barriers to accessing such support. Isolation appears to be shaped not only by stigma and limited peer networks, but also by the marginalisation of caregiver expertise within professional systems. This aligns with critiques in the IDD literature that highlight the frequent devaluation of experiential knowledge, which may contribute to disengagement from services and reduced help‐seeking.

### 1.3 Systemic Barriers and Fragmentation of Services

A recurring theme is the fragmentation of services for families managing complex behaviours like pica. Such fragmentation may pose direct safety risks, especially when interventions are delayed due to restrictive eligibility criteria or long waiting lists (Rosenberg et al. 2011). Such delays are not merely inconvenient, but may elevate risk, positioning families in prolonged states of unsupported crisis. This underscores the limitations of service models that are not designed to respond to behaviours requiring immediate and sustained risk management. Service gaps contribute to a diffusion of responsibility across systems, whereby families are repeatedly referred between professionals without clear ownership of care (Baker et al. 2021). Such experiences may be understood through the lens of systemic disorganisation, in which the intersection of medical, behavioural, and educational domains results in ambiguity regarding roles and accountability. This fragmentation not only undermines intervention effectiveness but may also erode caregiver trust in services over time.

Importantly, the absence of trauma‐informed care across systems emerges as a critical concern. Trauma‐informed frameworks emphasise safety, trustworthiness, collaboration, and empowerment; however, participants' accounts suggest that these principles are inconsistently applied in practice. Baker et al. (2021) highlight that caregivers of individuals with IDD may experience complex trauma linked not only to caregiving demands but also to repeated experiences of systemic failure. Service interactions themselves may function as sources of secondary stress or re‐traumatisation when families feel dismissed, unsupported, or excluded from decision‐making processes. Integrating trauma‐informed principles into disability services may therefore be essential, not only to support caregiver wellbeing but to enhance engagement and intervention sustainability.

Despite a growing body of clinical research on pica, the lived experiences of family caregivers remain critically underrepresented. The current study contributes to addressing this gap by foregrounding caregiver perspectives and highlighting the complex interplay between behavioural risk, psychological burden, and systemic constraints. Without such perspectives, intervention models risk remaining narrowly focused on behaviour reduction, without adequate consideration of feasibility, sustainability, or the broader caregiving context. Understanding how caregivers interpret, manage, and adapt to pica‐related challenges is therefore critical to the development of responsive, compassionate, and effective systems of support.

## 2 Methods

Interpretative Phenomenological Analysis (IPA) was employed because the study sought to explore not only caregivers' experiences of supporting a family member who engaged in pica, but also how they made sense of those experiences. In contrast to descriptive phenomenological approaches, which aim to stay close to the description of experience, IPA is interpretative and idiographic, making it well suited to exploring complex, emotionally charged, and personally significant experiences in depth (Smith et al. 2009; Smith et al. 2012).

### 2.1 Participants

Participants were recruited through a national organisation that supports families of individuals with intellectual disabilities. Study information was circulated via the organisation, and interested individuals contacted the researcher directly. Inclusion criteria were adults aged 18 years or over who were primary caregivers of an individual formally diagnosed with an intellectual disability who engaged in current or recent (within the last year) pica behaviour, and who were willing to take part in an in‐depth interview.

A purposive sampling strategy was used to recruit participants able to provide detailed, first‐person accounts of the caregiving experience. Four participants were recruited, all of whom were mothers. Although fathers and other caregivers were not excluded, mothers were the individuals who responded to the study invitation. This sample size was considered appropriate for IPA (e.g., Smith et al. 2009), which prioritises idiographic depth and detailed case‐by‐case analysis over breadth. Such numbers are typical in studies using IPA and are guided by epistemological commitments (Vasileiou et al. 2018). Participants ranged in age from 33 to 64, and their family members ranged in age from 5 to 36 years, including two males and two females. Participants were located across different regions of the UK.

### 2.2 Data Collection

Data were collected through semi‐structured interviews (see Table 1), an approach consistent with the idiographic and exploratory focus of Interpretative Phenomenological Analysis (IPA) (Smith et al. 2012). Participants were offered a choice between face‐to‐face and online interviews; all opted to be interviewed online. Conducting interviews remotely enabled participants to engage from familiar and private environments, which was particularly important given the sensitive nature of the topic.

**TABLE 1:** Semi‐structured interview guide.

Question	Interview prompt
1	As you know, we are here today to discuss pica and its impact. Before we do that, can you tell me a little more about your son or daughter? #
2	In your own words, how would you describe pica and your experience of its impact on your son/daughter? #
3	Could you tell me about when you first noticed pica behaviours? Prompt: How has this developed over time? #
4	Can you walk me through a day and night in your life, focusing on your experience in managing and navigating pica behaviours? #
5	What strategies or approaches have been most effective in managing these behaviours, and how did you come across them? # Prompt
6	What types of support or resources have been most helpful to your family in dealing with pica? #
7	Have you encountered any challenges in accessing certain types of support or resources? If so, what were they? Prompt: Were any resources not available, or any barriers to accessing them? #
8	How have you experienced the support you have received? Prompt: What, if anything, would have made it better? #
9	Reflecting on the emotional journey, what have been some significant challenges for you and your family? #
10	Can you share a particularly tough moment, and how did you find your way through it? Feel free to skip if this is too personal. #
11	How do you think others perceive pica, and what challenges or misunderstandings have you faced from society? #
12	What could help in improving understanding and support for families dealing with pica? #
13	Over time, how have your coping strategies evolved in managing the complexities of pica? #
14	Could you share a story where you felt a strong sense of achievement or resilience in dealing with pica? #
15	Looking ahead, what are your hopes for your child and their management of pica? #
16	What additional forms of support or resources would make a significant difference for your family and others in similar situations? #
Final Thoughts	
17	Is there anything else you'd like to share about your experience that we haven't discussed? #
18	What advice would you offer to other families navigating similar challenges? #

The interview schedule was developed in advance, guided by existing literature and refined through coproduction and consultation with a focus group comprising two professionals with lived experience of supporting a family member who engaged in pica, and two professionals with experience supporting individuals with behaviours of concern. Interviews ranged from approximately 50 min to 2.5 h. Three interviews were completed in a single sitting, while one was conducted across two sessions due to a technical disruption. All interviews were audio recorded and transcribed verbatim by the researcher. Transcripts were then prepared for analysis following guidance from Smith et al. (2012) and Peoples (2021). This involved the removal of non‐substantive utterances and repetitions where this did not alter meaning, while retaining the integrity and nuance of participants' original phrasing. Reflexive notes were maintained during data collection to support ethical sensitivity and to capture the researchers' reflections throughout the interview process.

This study employed Interpretative Phenomenological Analysis (IPA) to explore the lived experiences of family caregivers supporting individuals with intellectual disabilities who engage in pica. IPA is grounded in phenomenology, hermeneutics, and ideography (Smith et al. 2009) and is well suited to exploring how individuals make sense of complex, emotionally charged, and personally significant experiences. Quality is measured by the extent to which a compelling narrative is created, the focus on meaning‐making, thorough analysis and interpretation and attendance to convergence and divergence in participant accounts (Nizza et al. 2021).

An interpretative, rather than descriptive, phenomenological approach was adopted because the aim was not only to describe caregivers' experiences, but to explore how participants understood and made meaning of those experiences.

IPA prioritises depth over breadth, seeking to capture rich and detailed accounts from a small, purposively selected sample. This aligned with the aim of the study, which was to understand how caregivers interpreted and made meaning of their roles, responsibilities, and emotional experiences within the unique context of supporting a family member who engaged in a potentially life‐threatening behaviour.

Data were analysed using the six‐step approach to IPA outlined by Smith et al. (2009). Each transcript was read and re‐read several times to support immersion in the data. Initial exploratory comments were made on descriptive, linguistic, and conceptual aspects of each account, from which emergent themes were developed within individual cases. These themes were then examined for conceptual connections and organised into higher‐order groupings before the researcher moved to the next case, preserving IPA's idiographic commitment. Once all cases had been analysed, patterns of convergence and divergence across participants were explored to develop the final superordinate themes. Analytic decisions were documented throughout using annotated transcripts, theme development tables, and reflexive notes, providing an audit trail of how interpretations were reached and refined. Credibility was supported through repeated engagement with the data and on‐going reflexive scrutiny of interpretative decisions.

### 2.3 Ethical Considerations

Ethical approval was obtained from Tizard Centre Ethics Committee at the University of Kent, approval number 1076. Informed consent was obtained prior to participation, and all data were anonymised to protect participant identity. Due to the potentially distressing nature of the topic, participants were provided with information on counselling and support services following the interview. Safeguarding protocols were in place in case of disclosures indicating risk to the participant or the person they care for.

### 2.4 Researcher Reflexivity

Given the interpretative nature of IPA, reflexivity was maintained throughout data collection and analysis. The researcher's prior professional experience in disability support services brought both relevant sensitivity to the topic and the potential for preconceptions regarding caregiving, risk, and service responses. To minimise the imposition of these assumptions, reflective note taking was undertaken before, after interviews, and throughout analysis. The researcher also returned regularly to the original transcripts to ensure that developing interpretations remained grounded in participants' accounts rather than professional assumptions.

## 3 Results

Five superordinate themes were developed, each encompassing subthemes that reflect both shared experiences and individual nuance: Living on high alert; Systemic Failures in Care; Love in the Absence of Support; Emotional Erosion; Resourceful Parenting; The Silence Around Pica. Verbatim participant extracts are included throughout to preserve participants' voices and ground the analysis in lived experience. Interpretative commentary accompanies each theme, in line with IPA's double hermeneutic process: participants attempt to make sense of their own experiences. At the same time, the researcher sought to interpret how that sense‐making unfolds within their personal and relational worlds.

### 3.1 Theme 1—Living on High Alert

One of the most pervasive and emotionally resonant themes to emerge across all four participant accounts was the sense of living in a state of constant vigilance. Parents described being in a sustained state of readiness to protect their child from harm, with their routines, emotional well‐being, and family dynamics shaped around the relentless demands of managing pica. This unrelenting vigilance redefined how they experienced parenting. This theme encapsulates three interconnected subthemes: Hypervigilance, Guilt and Loss of Normal Life.

#### 3.1.1 Hypervigilance

Participants repeatedly emphasised the need for round‐the‐clock supervision,

Yeah, he's literally just started chipping off the walls again, and it's like I can't leave him for two minutes. (P3)

where the threat of ingestion was ever‐present and unpredictable,

*It is just the constant hypervigilance of the destruction that is made*. (P1)

necessitating continuous observation, even during periods typically associated with rest or respite.

*We… just can never relax in the house, and you know, even when she goes to bed, we can't relax because we have to watch the cameras*. (P4)

This state of hypervigilance had become normalised in daily life, yet its emotional cost was undeniable. While clinical frameworks may describe constant monitoring as a form of necessary supervision, for participants, this resulted in exhaustion that was not solely physical but stemmed from the psychological intensity of remaining perpetually alert, even when their child slept.

#### 3.1.2 Guilt

Intertwined with this vigilance was a profound and enduring sense of guilt. When incidents occurred, particularly those that required medical intervention, parents blamed themselves for not having done enough, even when the circumstances were beyond their control. They described going back over decisions they had made.

*I think when I let her go, I could have done what I always do and just followed…. I didn't. And then you think, I could have stopped it. So, you carry that with you*. (P2)

and feeling that they had not looked after their child well enough.

*When the doctor came back into the room and he looked at me, I thought I'd lost her. And in that moment, I thought, oh God, you know, why didn't I look after her… that was a tough moment for me*. (P4)

This guilt was not only retrospective but anticipatory, a constant emotional background noise to daily life.

#### 3.1.3 Loss of Normal Life

Hypervigilance and guilt are further compounded by the erosion of normality. Participants reflected on the absence of spontaneity, rest, and ordinary family life. Simple routines like watching television or going out with older children became compromised or impossible.

*We don't get to watch a film at night or anything… there's a camera sitting in front of me, and someone's always looking at it, you know*. (P4)

*It does put a stop on me doing a lot with the older kids, like going out for food or anything like that, … it's just, it's not doable*. (P3)

Where an activity was possible, it was not without sustained effort.

*His favourite things are going for walks in nature and sitting by water, and we can't do that anymore unless it's heavily controlled. Everything is managed*. (P1)

Whilst each individual issue might have been coped with, the extent and duration meant that families found it hard to remember what might be normal for other families.

*If there were any issues, you know, I can deal with that. But it's never just one thing. It's all day every day. No let‐up. You lose sight of what normal looks like*. (P2)

These losses extended beyond inconvenience. They fractured caregivers' sense of what family life should be. The emotional toll was not only the danger itself but the loss of intimacy and ordinary joy, an isolating difference from normative parenting experiences.

### 3.2 Theme 2—Systemic Failures in Care: Love in the Absence of Support

Participants described shortcomings in the support they received from healthcare and professional services, particularly when seeking help in life‐threatening situations. Experiences of being dismissed, misunderstood, or met with ignorance were widespread. This theme captures the structural and interpersonal breakdowns families encountered. Despite these failings, parents remained unwavering in their pursuit of safety, driven by love and moral obligation. Subthemes were identified: Medical Ignorance, Dismissal and Inappropriate Support.

#### 3.2.1 Medical Ignorance

Participants recounted repeated interactions with professionals who lacked basic awareness or understanding of pica.

*When she had swallowed some materials. One consultant was going round with his group of doctors doing the ward round, and he says this, young lady, I'm not really worried about what she swallowed, and I thought, oh, my God, I can't believe you just told all the doctors in the group that*. (P4)

In moments of acute danger, caregivers found themselves educating doctors and advocating for urgent intervention.

*I emailed the doctor's secretary and said, ‘We're on ward 10, no one's taking this seriously. ‘He came in that night…she's down for theatre tomorrow*. (P4)

These lapses in knowledge not only delayed care but also eroded trust in the systems designed to protect their children. Such moments illustrate the dual burden of caregiving: managing life‐threatening risk while also navigating professional ignorance.

#### 3.2.2 Trivialising

Participants reported experiences of being disregarded or not taken seriously by professionals, particularly in the early stages of seeking support

*Yeah. So, the consistent person that was there told me she thought it was funny ‘Oh she's got a thing for stones!’ and I was like, no, it's not funny. It's not a joke*. (P2)

Their concerns were often minimised or attributed to overreaction.

*So paediatrics, they told me it was mainly sensory. And I kept saying, no, it's not just that, it's dangerous. But they just weren't hearing it*. (P3)

This theme of dismissal reflects a broader issue of families feeling invalidated and unheard, compounding their emotional distress and delaying access to necessary care.

#### 3.2.3 Inadequate Support

Even when support was offered, participants frequently described it as poorly matched to families' needs.

*I went to our doctors. He referred me to our local hospital. I went to see a paediatrician there, and again…he told me to manage it, and it was like… I can't do any more than what I'm doing. I don't know how, and literally, that's it*. (P3)

Participants described unrealistic advice, superficial involvement and a lack of sustained engagement.

*You know, one nurse asked me why they didn't take all Lucy's teeth out, she says, ‘and that would stop her’*. (P4)

This systemic void pushed families into the role of care managers, coordinating safety plans, sourcing materials, and driving decisions without adequate input or guidance. Parents took on roles far beyond their remit because the consequence of not doing so was unthinkable.

### 3.3 Theme 3—Emotional Erosion

This theme captures the cumulative psychological strain of relentless caregiving, a gradual wearing down of emotional resilience shaped by fear, fatigue and a persistent sense of personal responsibility. Yet underpinning this erosion was a deep, unwavering love, driving parents to endure beyond their limits. Subthemes include Emotional Exhaustion, Coping Mechanisms and Hope versus Resignation.

#### 3.3.1 Emotional Exhaustion

Participants described chronic exhaustion stemming from sleepless nights, round‐the‐clock monitoring and relentless worry. This included the emotional impact,

*You know, I'd be making a dinner, she would have her hands in her pad, and poo would be everywhere. So emotionally it's very, very draining and very difficult for everybody*. (P2)

and extended beyond tiredness, manifesting in feelings of helplessness, frustration and at times despair.

*And just saying, I want to kill myself, and I know I don't, deep down, I know that I don't want to do that, and Karl needs me not to, but I'm afraid I'm just going to lose the capacity to see that's not what I want to do*. (P1)

Despite moments of hopelessness, participants pushed forward, not because they felt capable, but because there was no alternative.

*The coping strategies evolved because you don't really have a choice. You've got to cope. There's nothing else you can do*. (P4)

Several noted how this emotional exhaustion impacted their mental health, relationships and basic functioning, often in the absence of any structured support.

#### 3.3.2 Coping Mechanisms

In response to the on‐going emotional strain, families developed personal coping strategies, often improvised, self‐directed and born of necessity. These included immersion in nature during a short break,

*To be quite honest with you, I don't really have therapy or anything, but when the carer comes, the first thing I do is go for a big, long walk in the woods… no therapist can help me more than that*. (P4)

Reliance on other family members.

*To be honest I rely on me sister heavily for everything now*. (P3)

#### 3.3.3 Hope Versus Resignation

Caregivers described walking a tightrope between optimism and quiet acceptance, a tension shaped by repeated crises, slow progress, and a weight of uncertainty. While some held onto tentative hope for improvement

*I don't know. I live in hope that one day she'll stop doing it*. (P4)

others acknowledged a sense of resignation, a quiet acceptance that pica may always be a part of life.

*It is shit. It is truly shit. I mean, you know, it's relentless, but you do it. Because you love them*. (P1)

*… for us, it's just our life*. (P4)

This tension did not signal emotional failure, but an adaptive process: a form of psychological flexibility grounded in love. For these families, hope was not a naïve denial of difficulty, but a way to survive the present while still believing in something better.

### 3.4 Theme 4—Resourceful Parenting

Families showed extraordinary resourcefulness in protecting their loved ones. In the absence of professional guidance or adequate support, parents became adaptive problem‐solvers, drawing on creativity, intuition and deep emotional commitment. The theme encompasses two subthemes: Adaptive Strategies and Financial Strain.

#### 3.4.1 Adaptive Strategies

Parents described developing bespoke strategies to reduce harm, from custom‐made bedspreads to reinforced clothing, in response to the constant risk of ingestion and injury. These adaptations were often designed to prevent access to non‐food items, reduce choking risk during sleep, or protect the skin from compulsive biting or tearing.

*It's discovering that if I sew, like, two great big curtains together to make a bedspread… someone donating me an industrial sewing machine… people also offered me curtains… because she was eating the big clothes…*. (P4)

Extensive management strategies designed to keep their child safe.

*Literally, you manage to cover up things that will cause him harm…I've plastic sheets on pretty much every corner of the whole house, every window, anywhere, where he just could get to the plaster, causing him a lot of health problems*. (P3)

Simultaneously, in the absence of formal expertise, caregivers with lived experience turned to each other. Peer networks became vital spaces for shared knowledge, practical advice and emotional solidarity.

*Talking to other families that have the same problem, because they tell you what they have tried… maybe a family would tell me something they tried, and I would try it*. (P4)

Families were keen to both receive support and offer support to others.

*… I said, yes, I would speak to anybody where it will benefit people like my daughter and raise awareness*. (P2)

These adaptations and informal networks reflected more than practicality; they reflected a commitment to preserving their child's dignity, comfort and sense of identity. In sharing knowledge, families resisted isolation and affirmed the value of their experience.

#### 3.4.2 Financial Strain

The cost of innovation was steep. Many families described significant financial outlay on chew‐proof clothing, home modifications and safety equipment, often without reimbursement or formal support.

*We spent over …1000 pounds in one year, buying duvet covers. She was getting through a cover for nearly a night. It was a nightmare financially…the impact financially is quite serious as well*. (P4)

*Yes, so at the moment he is eating the wooden window frames, which is concerning because it's expensive to replace all the windows*. (P1)

The financial burden added another layer of stress to families already coping with high emotional and physical demands, often exacerbated by delays in accessing any statutory support.

*I think that if I hadn't had to fight for the EHCP, yeah, I mean in terms of my mental health, and also the enormous financial impact, we've spent 50,000 pounds on tribunals*. (P1)

For these parents, spending was not optional; it was an act of care, a means of safeguarding their child without external provision. The financial strain was not incidental but embedded in the systemic failure to provide timely, adequate, tailored support.

### 3.5 Theme 5—The Silence Around Pica

Despite its life‐threatening nature, pica remains largely invisible, rarely spoken about, poorly understood and socially minimised. Families described an enduring silence surrounding the behaviour: in medical conversations, in public discourse and even within personal networks. This theme captures the emotional toll of that silence through two subthemes: Isolation and Taboo and Call for Recognition.

#### 3.5.1 Isolation and Taboo

Participants consistently voiced feelings of isolation in their caregiving journey. The severity of pica was often misunderstood, even by close family and professionals, leaving caregivers feeling emotionally and practically alone.

*The consultants would say ‘never heard of pica, asking me ‘what is pica?*. (P4),

*I take him out, and yes, the thing is that people don't look at us. He's doing the weirdest things imaginable, and all the humans are acting like I just did not see that*. (P1)

Families described feeling judged, misunderstood or even ashamed when trying to explain their reality. For many participants, pica was shrouded in cultural and emotional taboo.

*I didn't know how to explain why he was chewing walls in the middle of the night…*. (P1).

*I feel more judged*. (P3)

This silence had consequences. The lack of understanding often left families feeling dismissed or unheard. When their experiences were minimised or ignored, seeking meaningful support or building understanding with others became harder.

#### 3.5.2 Call for Recognition

Despite, or perhaps because of this silence, participants expressed a clear and urgent desire for systemic and social recognition. Families called for greater awareness, specialist roles, and accurate recording of pica‐related risk.

*What external support and resources are there for pica? I've never seen any specialist roles or training*. (P3)

*I think there should be a network and support set up for families that have pica, because we could save our children's lives*. (P4)

This was not a cry for help; it was a deeply moral plea, rooted in love and a sense of responsibility. Families not only wanted recognition for themselves, but also a change that might protect others. In naming the silence, they became reluctant advocates, not because they sought the role but because no one else was speaking. For these families, silence is not benign; it is life‐threatening.

## 4 Discussion

This study set out to explore the lived experiences of family members supporting a person with intellectual disabilities who engages in pica. The findings offer a deeply emotive and contextually rich account of the complexities, burdens, and adaptations involved in this role. While each narrative was unique, shared patterns emerged across cases that illuminate both the private cost and the systemic neglect. The following analysis considers the meaning of these findings in relation to the literature and implications for theory, practice and research. Importantly, these findings are also usefully understood through a trauma‐informed care lens, which emphasises safety, trust, collaboration, empowerment, and the avoidance of re‐traumatisation (U.S. Department of Health and Human Services, Substance Abuse and Mental Health Services Administration, 2014; Harris and Fallot 2001).

### 4.1 Living With Risk: Hypervigilance and Existential Uncertainty

One of the most striking findings was the unrelenting proximity to life‐threatening danger. Caregivers described a state of existential hypervigilance, a heightened alertness beyond typical parental concern. Unlike risks that can be mitigated with supervision alone, pica presented a constant and unpredictable threat, where ingestion could occur in seconds with potentially fatal consequences. This persistent state of alertness may align more closely with trauma‐related processes than with traditional stress models echoing Schulz and Sherwood's (2008) work on chronic caregiving strain in high‐risk contexts, linking persistent alertness with negative health outcomes. Similarly, McConnell and Savage (2015) and Baker et al. (2021) identify ‘trauma‐like vigilance’, wherein caregivers remain in a state of anticipatory threat. In this study, hypervigilance shaped every aspect of daily life, from sleep patterns to routines, disrupting not just activity but a basic sense of safety.

For families supporting a loved one with pica, caregiving became defined not only by responsibility but by preparedness, fear, and sacrifice. Hypervigilance in this context was not a pathological symptom but a pragmatic and on‐going necessity, shaped by continuous risk. It was a state that restructured how families lived, related, and sustained themselves.

### 4.2 The Weight of Responsibility: Performing Every Role

Participants consistently described assuming multiple roles beyond traditional caregiving, acting as behavioural specialists, crisis responders, researchers and medical advocates. This ‘role creep’ reflects what McKendry and Baker (2018) identify as the growing expectation for families of individuals with a member who engages in pica to design and implement safety strategies in the absence of adequate professional support. A key aspect of this role expansion was epistemic labour: caregivers often found themselves educating professionals, even in medical emergencies, despite not being recognised as credible sources of knowledge.

Such injustice was particularly evident in healthcare settings, where participants encountered professionals unfamiliar with pica or dismissive of its severity. These professionals' knowledge gaps reinforced the systemic fragmentation noted by Sloper (2004) placing the logistical and emotional burden squarely on families.

Participants also described developing their own interventions: sewing reinforced bedding, sourcing protective clothing and coordinating multi‐agency responses independently. These acts demonstrate what McKendry and Baker (2018) call the emergence of families as ‘de facto experts’ in the absence of coordinated or informed services. While these adaptations speak to extraordinary parental resilience and ingenuity, they also highlight a troubling systems failure, in which vital safety planning is left to those least resourced, at considerable emotional and financial cost.

### 4.3 Emotional Erosion and the Paradox of Strength

Caregivers vividly described a gradual erosion, a cumulative toll derived from chronic exhaustion, sustained vigilance, and repeated trauma. Despite this strain, participants framed their endurance as a necessity rather than a choice. This echoes Bayat (2007) and Windle (2011), who conceptualise resilience as adaptation amidst distress, often achieved through reframing, prioritising, or redefining one's role. Several participants expressed quiet resignation that pica may never resolve, yet remained fiercely protective, consistent with Neely‐Barnes et al. (2011) finding that caregivers shift from seeking change to sustaining stability.

Notably, this resilience did not emerge from engagement with formal services, but from lived necessity. Coping strategies include time in nature, structured routines and moment‐to‐moment survival echoing emotion‐focused approaches (Lakhani et al. 2025; Pottie and Ingram 2008); however, this capacity to endure came at a significant cost: poor health, strained relationships and emotional fatigue. These findings affirm King et al. (2006) caution that resilience should not be idolised, is not universal, nor is it evidence of sufficiency in support. Rather, it reflects a fragile on‐going negotiation between strength and vulnerability played out in isolation under the weight of persistent risk.

### 4.4 The Silence Around Pica: From Taboo to Advocacy

A pervasive silence surrounded participants' experiences of pica despite its life‐threatening nature; the behaviour is often hidden, minimised, or met with discomfort in both professional and social settings. This mirrors wider patterns of health‐related stigma, where behaviours perceived as dangerous, uncontrollable or socially deviant are marginalised (Scambler 2009).

Caregivers described being judged or dismissed when discussing their child's behaviour, particularly in public. Their experiences reflect Fricker's (2007) concept of epistemic injustice, where caregivers' knowledge is discounted due to assumptions about their credibility. Left unsupported and invalidated, families navigated extreme risk with limited societal or institutional recognition. McKendry and Baker (2018) similarly note that the absence of clinical understanding often results in dismissive or inappropriate professional responses, reinforcing the invisibility of the condition.

This silence also constrained peer support; some participants hesitated to join parent groups or disclose experiences, fearing judgement or trivialisation, consistent with Bigby and Beadle‐Brown's (2018) findings on stigma and disengagement. In response, participants voiced a strong call for recognition: the inclusion of pica in professional training, national care frameworks and the development of specialist roles akin to those in epilepsy or autism services. These recommendations align with literature calling for integrated, condition‐specific supports that validate lived expertise and reduce fragmentation (Hagopian et al. 2011; Rosenberg et al. 2011).

Crucially, families did not see themselves solely as service users but as advocates. Their testimony was both a plea and a warning: without recognition, risk is compounded. As one participant stated, ‘I think some kids have died because of this, and it was never written down as pica’.

This theme affirms that silence is not neutral but a form of harm. For these families, invisibility equates to vulnerability. Their insights demand a cultural and structural shift that dismantles stigma, centres caregiver expertise, and treats pica with the seriousness it warrants.

### 4.5 Relational Commitment and Meaning Making

Across all five superordinate themes, a persistent thread emerged: caregiving was deeply rooted in love, protection, and relational duty. While not always explicitly named, this emotional bond underpinned the vigilance, creativity, advocacy, and endurance described by participants. Whether hand‐sewing chew‐proofing bedding or persevering through despair, caregivers framed their role through commitment, an identity shaped by connection and moral responsibility. As one parent expressed, ‘You do it because you love them, there isn't a choice’.

This aligns with research showing that caregivers of individuals with intellectual disabilities often construct meaning through love and obligation (Bayat 2007; Kimura and Yamazaki 2013). In high‐stress contexts, relational commitment can buffer emotional erosion by providing a sense of purpose, though it often carries a personal cost. For participants, caregiving was not merely a task but a way of being, an extension of loyalty and identity sustained by the parent–child bond.

### 4.6 Implications for Practice and Future Research

This study offers a rare, in‐depth account of family life shaped by the persistent threat of pica. The findings point to critical implications for practice and highlight areas where further research is urgently needed.

#### 4.6.1 Professional Training and Interdisciplinary Understanding

Participants' accounts revealed systemic failures rooted in professional misunderstanding or minimisation of pica. Consistent with McKendry and Baker (2018) and Hagopian et al. (2011), practitioners across health, education and social care were often unprepared to respond effectively. This was especially critical for emergency department and medical professionals, who are frequently the point of contact during pica‐related crises. Training must prioritise equipping clinicians, including emergency and paediatric staff, with knowledge of pica's risks and acute management. Beyond clinical awareness, professional development should also address the emotional and relational toll on families, incorporating lived experiences of risk, hypervigilance and caregiving burden to foster more attuned and empathetic care.

#### 4.6.2 Holistic and Sustained Support

Services must move beyond the short‐term, behaviour‐focused interventions and reflect the chronic, life‐altering nature of caregiving. This includes building holistic systems of support that integrate emotional support alongside behavioural strategies. Participant narratives highlight the need for family‐inclusive planning and sustained engagement, ensuring caregivers' mental health and resilience are supported over time.

#### 4.6.3 Valuing Caregiver Expertise

Caregivers described acting as advocates, engineers and crisis managers, a role that often yielded more effective solutions than formal guidance. This supports growing recognition that families hold vital experiential knowledge (Kirk and Glendinning 2004). Caregivers should be treated as co‐designers of interventions. Collaborative models that honour the lived expertise may enhance safety planning and service effectiveness.

#### 4.6.4 Service Design and Policy Recommendations

Findings support the need for a national framework on pica, akin to those for other high‐risk conditions, for example, the NICE Guidelines (National Institute for Health and Care Excellence 2012) or the use of psychotropic medication in intellectual disability (STOMP/STAMP) (NHS England 2019). Such frameworks provide guidance, training standards, and risk management protocols, elements currently lacking for families managing pica. Such frameworks could include explicit care pathways, condition‐specific guidance, and dedicated roles. As Turnbull et al. (2015) argue, effective systems are family‐centred, flexible, and context‐responsive.

Taken together, these findings suggest that integrating trauma‐informed care into intellectual disability services is not optional but essential in the context of pica. Such an approach would extend beyond behaviour management to address relational safety, caregiver wellbeing, and systemic responsiveness. Embedding trauma‐informed principles may enhance engagement, reduce distress, and improve the sustainability of interventions across both clinical and home environments (U.S. Department of Health and Human Services, Substance Abuse and Mental Health Services Administration, 2014; Harris and Fallot 2001).

#### 4.6.5 Future Research Directions

Further research is warranted to broaden the current focus beyond mothers, incorporating the perspectives of fathers, siblings, and extended family members. Moreover, given the substantial demands placed on family carers, many families may ultimately seek residential care for their relative. This shift introduces a distinct caregiving context, characterised by new emotional, relational, and practical dynamics, which also merit exploration to ensure the voices of all family members are adequately represented. Longitudinal studies could provide valuable insights into how caregiving experiences and needs evolve across key life transitions, such as adolescence, adulthood, and placement into residential care. Crucially, research must continue to centre family voices, especially those supporting individuals with complex or marginalised profiles.

#### 4.6.6 Limitations

While this study offers important insights into the lived experiences of family members supporting an individual with intellectual disabilities who presents with pica, several limitations must be acknowledged. First, the sample was small and homogeneous. Although this is consistent with the idiographic aims of Interpretative Phenomenological Analysis (Smith et al. 2012), it limits the range of perspectives captured. All participants were mothers, and all were recruited through a support network, which may have introduced self‐selection bias. This may limit the theoretical transferability of understandings to other contexts. For example, the findings from this study may not reflect the experiences of fathers, siblings, or families who are less connected to support services. In addition, all participants were supporting their child at home, and these experiences may differ from those of families or carers in residential or institutional contexts.

A further limitation is that the study captures caregiver perspectives only and does not include the voices of individuals who themselves engage in pica. Although direct participation may be more difficult in some cases because of cognitive or communication needs, the absence of these perspectives remains important, and future research should seek to include them where feasible. Finally, although the age range of the individuals supported was broad, the small sample and idiographic design did not allow for comparison across developmental stages or age groups. The findings should therefore be understood as situated, interpretative accounts, offering an in‐depth contribution to the emerging understanding of pica and family caregiving.

## 5 Conclusion

This study set out to explore the lived experiences of family members supporting a person with intellectual disabilities who engages in pica. Illuminating the intense psychological, emotional and practical toll of managing a behaviour that is poorly understood, inconsistently supported and profoundly dangerous.

Five superordinate themes emerged: Living on High Alert, Systemic Failures in Care, Emotional Erosion, Resourceful Parenting and The Silence Around Pica. Together, these themes revealed a caregiving role defined not only by constant supervision but also by emotional labour, ingenuity and advocacy in the face of systemic neglect. Participants described enduring hypervigilance, guilt, isolation and exhaustion, yet also demonstrated extraordinary resilience through peer learning, creative adaptations, advocacy and deep love.

## Funding

The authors have nothing to report.

## Ethics Statement

The University of Kent's Tizard Centre Research Ethics Committee granted ethical approval for the study (reference 1076).

## Conflicts of Interest

The authors declare no conflicts of interest.

## Data Availability

The data that support the findings of this study are available on request from the corresponding author. The data are not publicly available due to privacy or ethical restrictions.
